# Sarcopenic Obesity in People With HIV: Diagnostic Criteria and Classification—A Scoping Review With a Meta‐Analysis of Prevalence

**DOI:** 10.1155/jobe/7991603

**Published:** 2026-08-04

**Authors:** Igor Massari Correia, Chimenny Auluã Lascas Cardoso De Moraes, Márcio Fernando Tasinafo Júnior, Joana Brilhadori, Jeferson Roberto Collevatti Dos Anjos, Euripedes Barsanulfo Gonçalves Gomide, Lisa Fernanda Mazzonetto, Alcivandro De Sousa Oliveira, Lara Souza Silva, Giovana Finco, Kelly K. O. ’Brien, Kristine M. Erlandson, Alisson R. Webel, Vitor Oliveira, Jéssica Fernanda Corrêa Cordeiro, André Pereira Dos Santos

**Affiliations:** ^1^ Ribeirão Preto School of Nursing, University of São Paulo, Ribeirão Preto, Brazil, usp.br; ^2^ Study and Research Group on Anthropometry, Training, and Sport, School of Physical Education and Sport of Ribeirão Preto, University of São Paulo, Ribeirão Preto, Brazil, usp.br; ^3^ School of Physical Education and Sport of Ribeirão Preto, University of São Paulo, Ribeirão Preto, Brazil, usp.br; ^4^ Claretiano-University Center, Batatais, Brazil; ^5^ Department of Physical Therapy, Temerty Faculty of Medicine, University of Toronto, Toronto, Canada, utoronto.ca; ^6^ Institute of Health Policy, Management and Evaluation, Dalla Lana School of Public Health, University of Toronto, Toronto, Canada, utoronto.ca; ^7^ Rehabilitation Sciences Institute, University of Toronto, Toronto, Canada, utoronto.ca; ^8^ University of Colorado Anschutz Medical Campus, Aurora, Colorado, USA, ucdenver.edu; ^9^ Department of Child, Family, and Population Health Nursing, University of Washington, Seattle, Washington DC, USA, washington.edu; ^10^ Department of Biobehavioral Nursing and Health Informatics, University of Washington, Seattle, Washington DC, USA, washington.edu; ^11^ National Council for Scientific and Technological Development (CNPq), Brasília, Brazil; ^12^ STAHR Scholar, University of California, San Diego, California, USA, berkeley.edu; ^13^ International AIDS Society, Geneva, Switzerland

**Keywords:** HIV, sarcopenic obesity, scoping review

## Abstract

**Objectives:**

(a) To describe studies that evaluated sarcopenic obesity (SO) in people with HIV (PWH); (b) to summarize the methods and diagnostic criteria used to define SO in PWH; and (c) to estimate the overall prevalence of SO in this population.

**Design:**

Scoping review conducted according to established methodological frameworks and reported following PRISMA‐ScR recommendations.

**Setting and Participants:**

Original observational studies conducted in clinical or research settings involving adults (≥ 18 years) with HIV that explicitly applied diagnostic criteria for SO were included.

**Methods:**

Studies were identified through searches in 12 electronic databases from inception to August 2024, without language restrictions. The scoping review followed five stages: identification of the research question, identification and selection of relevant studies, data extraction, and synthesis of findings. Diagnostic approaches for SO included body composition assessed by dual‐energy X‐ray absorptiometry (DXA) or computed tomography (CT), anthropometric measures such as body mass index (BMI), and functional assessments including handgrip strength and the Short Physical Performance Battery (SPPB). An exploratory random‐effects meta‐analysis was performed to estimate the overall prevalence of SO in PWH.

**Results:**

Three observational studies were included, two from Italy and one from the United States, comprising 2873 participants, predominantly male (*n* = 2174 [76%]). Mean age ranged from 47 to 52 years, and mean duration of HIV diagnosis and antiretroviral therapy exceeded 10 years. Seven distinct diagnostic criteria for SO were identified, with DXA being the most frequently used method. The prevalence of SO ranged from 0.76% to 20.8%, and the pooled overall prevalence was 8.18%.

**Conclusions and Implications:**

SO remains insufficiently explored among PWH. The wide variability in diagnostic criteria and assessment methods contributes to inconsistent prevalence estimates, underscoring the need for standardized, population‐specific diagnostic approaches.

## 1. Introduction

The widespread use of antiretroviral therapy (ART) has transformed HIV infection into a chronic condition and markedly increased life expectancy among people with HIV (PWH) [1–3]. As survival improves, this population is experiencing progressive aging. Importantly, accumulating evidence suggests that PWH are affected not only by chronological aging but also by features of accelerated biological aging, influenced by persistent inflammation, immune dysregulation, and long‐term exposure to ART. In this context, age‐related alterations in body composition may occur earlier and more intensely in PWH than in the general population [4–6].

These changes contribute to the growing burden of obesity and more pronounced declines in muscle mass, strength, and physical function compared with people without HIV, even after adjustment for age and comorbidities [5, 7–12]. Together, these unfavorable and concurrent changes in adiposity and muscle health create a biological and clinical context that may predispose PWH to sarcopenic obesity (SO), influenced by ART‐related metabolic effects, excessive caloric intake, and sedentary behavior. SO is associated with an increased risk of chronic diseases, functional impairment, and mortality, augmenting cardiovascular risk more than obesity or sarcopenia alone [7–9, 13–18].

SO represents a complex condition integrating components of both obesity and sarcopenia. Despite advancing diagnostic frameworks, substantial variability in assessment methods persists, with general population prevalence ranging from 0.1% to 48% depending on the criteria applied [13, 14, 19–21]. Among PWH, the identification of SO may be further complicated by HIV‐specific factors such as altered fat distribution, lipodystrophy, and ART‐associated weight gain, which may limit the applicability of diagnostic thresholds derived from HIV‐negative populations [5, 22–26].

In parallel, few studies have specifically evaluated methods or criteria for diagnosing SO in this population [27–29]. Consequently, the prevalence and clinical relevance of SO among PWH remains insufficiently characterized, which may affect the reliability of assessment methods, the identification of cases, and the estimation of prevalence in this population. Given the increasing longevity of this population and the absence of tailored diagnostic criteria, a synthesis of available evidence is warranted. Therefore, this study aimed to (a) describe studies evaluating SO in PWH, (b) summarize diagnostic methods and criteria, and (c) estimate the overall prevalence of SO in this population. By addressing these factors, this study may help clarify existing research gaps and support clinicians and researchers in applying available diagnostic criteria to better understand the prevalence and clinical implications of SO among PWH.

## 2. Methods

### 2.1. Study Design

This scoping review follows the JBI methodological framework and the PRISMA‐ScR guidelines. The design was chosen to map heterogeneous diagnostic definitions and assessment methods for SO in PWH. The protocol is registered on the Open Science Framework (DOI: 10.17605/OSF.IO/MRZKG) [30–33].

### 2.2. Identification of the Research Question

Guided by the Population, Concept, and Context (PCC) framework, the primary question was “What diagnostic criteria and assessment methods have been used in the literature to identify SO in adults with HIV?” [34]. Given the exploratory nature of a scoping review, no restrictions were imposed regarding specific assessment methods or cutoff points, allowing the inclusion of heterogeneous diagnostic approaches. Although prevalence was not defined as a primary eligibility criterion, studies reporting prevalence estimates of SO among PWH were included when available. When prevalence data were not explicitly reported, the corresponding authors were contacted to obtain this information. Only one author needed to be contacted, and the requested data were successfully obtained [33].

### 2.3. Identification of Relevant Studies

The database search was conducted in April 2024. To ensure reproducibility, the reviewers were required to retrieve the same number of records from each database using identical search strategies. The descriptors used in this research were selected through the databases “Health Sciences Descriptors” (DeCS) and “Medical Subject Headings” (MESH). No filters, language restrictions, or publication periods were used in the search (i.e., databases were searched from inception).

The following electronic databases were searched from inception: Excerpta Medica DataBase (EMBASE), PubMed, Scopus, Web of Science, Latin American and Caribbean Health Sciences Literature (LILACS), EBSCO, Virtual Health Library (BVS), Cochrane Central Register of Controlled Trials, and Physiotherapy Evidence Database (PEDro). In addition, MedRxiv and Google Scholar were searched to identify relevant gray literature. To ensure the inclusion of recently published and emerging evidence, the search was updated on August 10, 2024, and all eligible studies published up to this date were considered for inclusion. The full search strategies used for each database and repository, as well as the number of records retrieved, are presented in Supporting Table 2.

### 2.4. Selection of the Studies

Eligible studies included intervention, cross‐sectional, and observational designs involving adults (≥ 18 years) with HIV. Reviews, editorials, and conference abstracts were excluded. Selection occurred in two phases (title/abstract and full text) by two independent reviewers, with a third reviewer adjudicating any discrepancies.

### 2.5. Data Extraction and Description of the Data

Identified studies were exported to EndNote Basic to eliminate duplications. Nonduplicated studies were exported to Rayyan software for review [35, 36]. Additionally, a manual search was conducted in the reference lists of the studies selected to identify other eligible studies. Prior to formal data extraction, reviewers conducted a calibration exercise using a subset of studies to ensure consistency in data interpretation and extraction. Data were then independently extracted by pairs of reviewers using standardized forms [32].

### 2.6. Collating Data Analysis and Synthesis of the Data

Data were organized into characterization tables including general study information (author, year, and country), participant characteristics (demographics and HIV clinical variables), assessment methods, and SO prevalence. To explore prevalence variability across different criteria, a quantitative synthesis was performed using a random‐effects model in Comprehensive Meta‐Analysis (CMA) software (Version 4). Heterogeneity was assessed via Cochran’s *Q* and I^2^ statistics, with 95% confidence intervals (95% CIs) reported for all estimates.

## 3. Results

**FIGURE 1:**
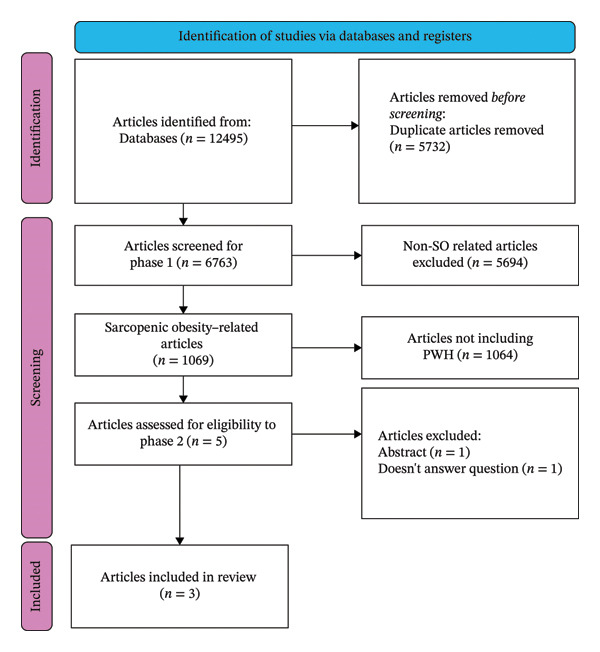
Flow diagram (PRISMA). *Notes*: extraction table of the included studies; Phase 1: reading titles and abstracts and Phase 2: complete reading of the article.

The database search identified a total of 12,495 records. After the removal of duplicates, 6763 studies remained for title and abstract screening. Following this phase, five full‐text articles were assessed for eligibility. Three studies met the inclusion criteria and were included in the final synthesis. The study selection process and reasons for exclusion are presented in the PRISMA flow diagram (Figure 1) [32].

The three included studies were published between 2023 and 2024 and conducted in high‐income countries (two in Italy and one in the United States). All studies had an observational design. Sample sizes ranged from 187 to 2379 participants, totaling 2873 PWH. Across studies, participants were predominantly male (approximately 76%), with mean ages ranging from 47 to 52 years. The mean duration since HIV diagnosis and time on ART exceeded 15 years in all studies (16–21 years) Table 1.

**TABLE 1:** Characteristics of participants with HIV in the included studies.

Articles	Milic [29] *n* total = 2379	De Vincentis [28] *n* total = 307	Swartz et al. [30] *n* total = 187
Male *n* (%)	1707 (72%)	307 (100%)	160 (86%)
Mean age in years ± range	52 ± 10^∗^	47 ± 5.3^∗∗^	51.2 ± 12.3^∗∗^
Mean CD4 count in cells/mm^3^ ± range	712 ± 366^∗^	NR	807 ± 330^∗∗^
Mean time since HIV diagnosis in months ± range	254 ± 176^∗^	196.8 ± 104.4^∗∗^	195.6 ± 115.2^∗∗^
Mean time under ART in months ± range	NR	165.6 ± 94.8^∗∗^	NR
Mean weight in kg ± range	71 ± 17^∗^	NR	99.0 ± 18.5^∗∗^
Mean ASMI in kg/m^2^ ± range	7.12 ± 1.70^∗^	7.8 ± 1.7^∗∗^	NR
Mean TSMI in cm^2^/m^2^ ± range	NR	NR	61.6 ± 10.8^∗∗^
Mean ALM/W in % ± range	NR	32.7 ± 7.5^∗∗^	NR
Mean BMI in kg/m^2^ ± range	24.1 ± 4.5^∗^	23.9 ± 3.1^∗∗^	32.6 ± 6.3^∗∗^
Mean VAT in cm^2^ ± range	147 ± 114^∗^	NR	229.7 ± 104.4^∗∗^
Mean BF in % ± range	NR	24.6 ± 6.3^∗∗^	NR
Mean SPPB score ± range	12.0 ± 1.0^∗^	NR	NR
Low HGS *n* (%)	538 (39%)	NR	NR
Country	Italy	Italy	United States
Study design	Observational, longitudinal study	Cross‐sectional observational study	Cross‐sectional observational study
Main aim	Describe prevalence, incidence, and risk factors for SO phenotypes in PWH and their association with subclinical cardiovascular disease	To prospectively explore the relationship between serum sex steroids, body composition, and bone health status in men with HIV	Use CT imaging to identify obesity phenotypes in PWH

*Note:* Antiretroviral therapy (ART); interquartile range (IQR); calculation of appendicular lean mass in kg/(height in m)^2^); calculation of muscle mass area in cm^2^/(height in m)^2^); Short Physical Performance Test Battery (SPPB); handgrip strength (HGS); low HGS: PWH who presented HGS below the cutoff point; computerized tomography (CT).

Abbreviations: ALM/W, appendicular lean mass/weight; ASMI, appendicular skeletal muscle index; BF, body fat; BMI, body mass index; NR, not reported; SD, standard deviation; TSMI, total skeletal muscle index; VAT, visceral adipose tissue.

^∗^Interquartile.

^∗∗^Standard deviation.

Seven distinct diagnostic criteria for SO were identified across the included studies. Body composition assessment methods varied substantially and included dual‐energy X‐ray absorptiometry (DXA), computed tomography (CT), and anthropometric measures such as body mass index (BMI). Muscle strength was primarily assessed using handgrip strength (HGS), while physical function was evaluated using instruments such as the Short Physical Performance Battery (SPPB). DXA was the most frequently used method for assessing body composition. Appendicular skeletal muscle index (ASMI) and BMI were the most common criteria for diagnosing SO (Table 2).

**TABLE 2:** Diagnostic criteria for SO in PWH.

Article	Sarcopenia diagnosis	Obesity diagnosis	SO diagnosis
Milic [29]	HGS (Jamar Hand Dynamometer)	BMI (DXA)	Low HGS + High VAT
	Cutoff points according to Wang et al. [37])	Cutoff ≥ 30 kg/m^2^	
			Low HGS + High BMI
		VAT (CT or DXA) cutoff ≥ 160 cm^2^	
			Low ASMI + High VAT
	SPPB		
	Cutoff < 11		
			Low ASMI + High BMI
	ASMI (DXA)		
	Cutoff < 7.26/< 5.45 kg/m^2^ for males/females		

De Vincentis [28]	DXA	DXA	Low ASMI + High BMI and/or % BF
	Cutoff	Cutoff BMI ≥ 30 kg/m^2^ and/or % BF (> 26% for males 20–39 years old and > 28% for 40–50 years old)	Low ASM/W + High BMI and/or % BF
	ASMI < 7.26		
	cutoff		
	ASM/W < 28.27%		

Swartz et al. [30]	N/A	N/A	There was an isolated diagnosis of SO using CT as a diagnostic method SO was defined as TSMI < 43 and BMI < 25 kg/m^2^ or TSMI < 53 and BMI ≥ 25 kg/m^2^ for males and TSMI < 41 for females

*Note:* Handgrip strength (HGS); Short Physical Performance Test Battery (SPPB): calculation of appendicular lean mass in kg/(height in m)^2^); calculation of muscle mass area in cm^2^/(height in m)^2^); dual‐energy X‐ray absorptiometry (DXA). The term “low” used in the “SO diagnostic criteria” column refers to values below the cutoff point for diagnosing SO and the term “high” means above the cutoff point for diagnosing SO.

Abbreviations: ASMI, appendicular skeletal muscle index; ASM/W, appendicular lean mass/weight; BF, body fat; BMI, body mass index; CT, computed tomography; N/A, not available; SO, sarcopenic obesity, TSMI, total skeletal muscle index; VAT, visceral adipose tissue.

The reported prevalence of SO among PWH varied markedly according to the diagnostic criteria applied, ranging from 0.76% to 20.8%, with the highest prevalence (20.8%) based on low ASMI + high VAT and the lowest prevalence based on low ASMI + high BMI (Table 3).

**TABLE 3:** Prevalence of SO in PWH across the included studies.

Articles	Milic [29]				De Vincentis [28]		Swartz et al. [30]
Diagnostic criteria	Low HGS + high VAT	Low HGS + high BMI	Low ASMI + high VAT	Low ASMI + high BMI^∗^	Low ASMI + high BMI and/or % BF^∗^	Low ASM/W + high BMI and/or % BF	Low TSMI
Prevalence (*n*)	19.7% (*n* = 243 of 1236)	3.6% (*n* = 79 of 2183)	20.8% (*n* = 170 of 817)	0.76% (*n* = 17 of 2224)	11.4% (*n* = 35 of 307)	12.4% (*n* = 38 of 307)	11% (*n* = 20 of 187)

*Note:* Handgrip strength (HGS); ASMI: calculation of appendicular lean mass in kg divided by height in meters squared; TSMI is defined as muscle mass area (cm^2^) divided by height squared (m^2^).

Abbreviations: ASMI, Appendicular Skeletal Muscle Index; ASM/W, appendicular lean mass/weight; BF, body fat; BMI, Body Mass Index; SO, sarcopenic obesity; TSMI, Total Skeletal Muscle Index; VAT, visceral adipose tissue.

^∗^Similar Diagnostic Criteria.

**FIGURE 2:**
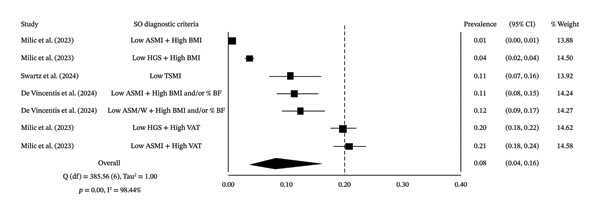
Meta‐analysis of the prevalence and overall prevalence of SO in PWH. *Notes*: Sarcopenic obesity (SO); handgrip strength (HGS); appendicular skeletal muscle index (ASMI: calculation of appendicular lean mass in kg/(height in m)^2^); total skeletal muscle index (TSMI: calculation of muscle mass area in cm^2^/(height in m)^2^); body mass index (BMI); visceral adipose tissue (VAT); appendicular lean mass/weight (ASM/W); body fat (BF); ^∗^Similar diagnostic criteria, degrees of freedom (df).

When prevalence estimates were pooled using a random‐effects model, the overall prevalence of SO was 8.18% (95% CI: 4.02%–15.9%), representing 235 cases among 2873 participants. Heterogeneity across studies was high (I^2^ value of 98.44% and a Q value of 385.56; df = 6), reflecting substantial methodological and diagnostic variability (Tau [2] = 1.00) (Figure 2). Rather than reflecting true epidemiological differences, the wide range of SO prevalence observed across studies appears to be largely driven by differences in diagnostic criteria and assessment methods.

## 4. Discussion

This scoping review synthesized the available evidence on the diagnostic criteria and reported prevalence of SO among PWH, highlighting substantial methodological variability across studies and its implications for prevalence estimates and clinical interpretation. Despite the growing clinical relevance of unfavorable body composition changes in this population, our findings demonstrate that SO remains insufficiently characterized in the literature, with only three eligible studies identified.

Our findings indicate substantial heterogeneity in SO identification, with seven distinct diagnostic approaches identified across three studies. This variability resulted in prevalence estimates ranging from 0.76% to 20.8% [27–29], highlighting the lack of standardized guidelines for PWH.

Currently, there are no specific guidelines for the diagnosis of SO in PWH. Furthermore, while SO guidelines for the general population recommend combining body fat percentage (%BF) and ALM/W with functional parameters, such as HGS, most diagnostic criteria applied to PWH rely solely on anthropometric indices (BMI) and body composition measures (ALMI, ALM/W, TSMI, VAT, and %BF) [38, 39]. The identification of SO in PWH is further complicated by HIV‐specific factors, such as altered fat distribution, lipodystrophy, and ART‐associated weight gain [5, 22–26]. Diagnostic thresholds derived from HIV‐negative populations may, therefore, have limited applicability in this context. In addition, differences in imaging techniques (e.g., DXA versus CT), functional assessments, and cutoff points for sarcopenia and obesity hinder direct comparisons across studies and limit the generalizability of prevalence estimates.

PWH represent a population uniquely exposed to factors that may accelerate unfavorable changes in body composition. Increased longevity, persistent immune activation, chronic inflammation, and long‐term exposure to ART may promote earlier accumulation of fat mass and declines in muscle mass, strength, and physical function compared with HIV‐negative individuals [5, 10–12, 40]. These concurrent processes, compounded by excessive food intake high in calories, saturated fats, added sugars, and sedentary behavior, create a biological context that may predispose PWH to SO at younger ages and with distinct clinical characteristics compared to the general population [7–9].

SO prevalence in the general population varies widely (0.8%–11.0%) depending on the criteria applied. While global SO prevalence in older adults (∼70 years) is approximately 11%, our synthesis found a comparable pooled prevalence of 8.18% in PWH despite a mean age 20 years younger (47–52 years) [14]. Notably, De Vincentis reported a 12.4% prevalence in young men with HIV—more than double that of HIV‐negative populations despite a 20‐year age gap [21, 27]. This disparity aligns with the accelerated aging phenotype in PWH, where immunological markers often resemble those of seronegatives 2 decades older. Thus, while ethnic, cultural, and geographic factors contribute to this variability, the accelerated aging phenotype in PWH appears to be a key driver of the higher SO prevalence observed in this group [4].

Although scoping reviews traditionally avoid quantitative synthesis, we calculated an overall prevalence estimate to provide an aggregated view of the burden of SO among PWH. We acknowledge that pooling prevalence estimates derived from heterogeneous diagnostic criteria resulted in high statistical heterogeneity. However, rather than serving as a definitive epidemiological measure, this estimate should be interpreted as an illustrative summary that highlights the magnitude of variability and reinforces the need for standardized diagnostic approaches in future research.

This review has limitations that should be acknowledged. First, the small number of included studies (*n* = 3) and the predominance of male participants limit the generalizability of our findings, particularly concerning PWH in diverse demographic contexts. The reliance on male‐predominant samples is a critical factor, as sex differences significantly influence body composition and SO prevalence. Moreover, the included studies predominantly evaluated individuals with long‐standing HIV infection, with an average duration of more than 15 years ART and mean CD4 counts above 700 cells/mm^3^. This clinically stable profile may not reflect the broader heterogeneity of PWH, as both ART duration and time since diagnosis substantially influence body composition trajectories. Additionally, most studies included younger populations compared with typical SO research in geriatric cohorts. Methodological constraints also include a lack of adherence to current diagnostic recommendations in some studies and a restriction to high‐income countries, affecting external validity. Consequently, the high heterogeneity observed in our meta‐analysis likely reflects these differing diagnostic criteria, sample characteristics, ART‐related body composition changes, and regional variations rather than true population variability. Finally, although efforts were made to retrieve missing data by contacting authors, unpublished data or ongoing studies may not have been captured.

In conclusion, SO among PWH remains an underexplored and methodologically heterogeneous condition. The wide variability in diagnostic criteria and assessment methods limits accurate estimation of prevalence and complicates clinical interpretation. Future studies should prioritize larger, more diverse cohorts—including female populations—and focus on the development and validation of standardized, HIV‐specific diagnostic frameworks to improve identification, comparability, and clinical management in this growing population.

## Funding

This study was funded by Coordenação de Aperfeiçoamento de Pessoal de Nível Superior, Finance Code 001.

## Disclosure

All author and institution names have been anonymized in accordance with the journal’s guidelines.

## Conflicts of Interest

The authors declare no conflicts of interest.

## Supporting Information

Additional supporting information can be found online in the Supporting Information section.

## Supporting information


**Supporting Information** Supporting Information. This supporting material provides additional methodological and bibliographic information related to the scoping review presented in this manuscript. Supporting Table 1: PRISMA‐ScR Checklist: Completed checklist in accordance with the PRISMA Extension for Scoping Reviews (PRISMA‐ScR), indicating page and line numbers corresponding to the items reported in this manuscript. Items marked as “NA” indicate nonapplicability. This supporting material ensures the transparency, reproducibility, and reporting quality of the scoping review. Supporting Table 2: Search strategy and number of studies retrieved per database: This table details the search strategies, including terms used, databases consulted, search dates, and the number of studies retrieved, with breakdowns for aggregated databases (e.g., EBSCO and BVS).

## Data Availability

The data that support the findings of this study are available on request from the corresponding author. The data are not publicly available due to privacy or ethical restrictions.
