# Supplementary material for: Sarcopenic Obesity in People With HIV: Diagnostic Criteria and Classification—A Scoping Review With a Meta‐Analysis of Prevalence
Source: J Obes. 2026 Aug 4;2026:7991603. doi: 10.1155/jobe/7991603 (PMC13435694; doi:10.1155/jobe/7991603)
Supplement: Supplementary file 1 [file JOBE-2026-7991603-s001.docx]

**Supplementary Material Description**

This supplementary material provides additional methodological and bibliographic information that supports the scoping review presented in the main manuscript. It includes detailed information on the literature search strategy and a completed PRISMA-ScR checklist to ensure methodological transparency and reporting quality.

Supplementary Table 1 – PRISMA-ScR Checklist: Checklist indicating compliance with the Preferred Reporting Items for Systematic reviews and Meta-Analyses extension for Scoping Reviews (PRISMA-ScR), with the corresponding page and line references in the manuscript. Items marked "NA" were not applicable to this review.

Supplementary Table 2 – Search strategy and number of studies retrieved per database:
Summary of the number of articles retrieved from each scientific database using the descriptor "Sarcopenic obesity" (and its equivalents in Portuguese and Spanish, where applicable). The search was conducted in April 2024, and the specific day is indicated. For aggregated databases such as EBSCO and BVS, the number of records per sub-database is also detailed.

These supplementary materials are essential for ensuring reproducibility of the review methods and transparency in reporting, as well as for readers seeking additional details on the search and reporting framework.

**Supplementary Table 1-** Preferred Reporting Items for Systematic reviews and Meta-Analyses extension for Scoping Reviews (PRISMA-ScR) Checklist

| **SECTION** | **ITEM** | **PRISMA-ScR CHECKLIST ITEM** | **REPORTED ON PAGE #** |
| --- | --- | --- | --- |
| **TITLE** | | | |
| Title | 1 | Identify the report as a scoping review. | Page 1 (line 1-2) |
| **ABSTRACT** | | | |
| Structured summary | 2 | Provide a structured summary that includes (as applicable): background, objectives, eligibility criteria, sources of evidence, charting methods, results, and conclusions that relate to the review questions and objectives. | Page 1-3 (line 4-52) |
| **INTRODUCTION** | | | |
| Rationale | 3 | Describe the rationale for the review in the context of what is already known. Explain why the review questions/objectives lend themselves to a scoping review approach. | Page 3-5 (line 52-102) |
| Objectives | 4 | Provide an explicit statement of the questions and objectives being addressed with reference to their key elements (e.g., population or participants, concepts, and context) or other relevant key elements used to conceptualize the review questions and/or objectives. | Page 5-6 (line 104-125) |
| **METHODS** | | | |
| Protocol and registration | 5 | Indicate whether a review protocol exists; state if and where it can be accessed (e.g., a Web address); and if available, provide registration information, including the registration number. | Page 6 (line 127-142) |
| Eligibility criteria | 6 | Specify characteristics of the sources of evidence used as eligibility criteria (e.g., years considered, language, and publication status), and provide a rationale. | Page 6-7 (line 144-155) |
| Information sources* | 7 | Describe all information sources in the search (e.g., databases with dates of coverage and contact with authors to identify additional sources), as well as the date the most recent search was executed. | Page 7 (line 157-170) and Supplementary material. |
| Search | 8 | Present the full electronic search strategy for at least 1 database, including any limits used, such that it could be repeated. | Supplementary material. |
| Selection of sources of evidence† | 9 | State the process for selecting sources of evidence (i.e., screening and eligibility) included in the scoping review. | Page 7-8 (line 172-184) |
| Data charting process‡ | 10 | Describe the methods of charting data from the included sources of evidence (e.g., calibrated forms or forms that have been tested by the team before their use, and whether data charting was done independently or in duplicate) and any processes for obtaining and confirming data from investigators. | Page 8-9 (line 186-200) |
| Data items | 11 | List and define all variables for which data were sought and any assumptions and simplifications made. | Page 9 (line 202-213) |
| Critical appraisal of individual sources of evidence§ | 12 | If done, provide a rationale for conducting a critical appraisal of included sources of evidence; describe the methods used and how this information was used in any data synthesis (if appropriate). | NA |
| Synthesis of results | 13 | Describe the methods of handling and summarizing the data that were charted. | Page 9 (line 213-222) |
| **RESULTS** | | | |
| Selection of sources of evidence | 14 | Give numbers of sources of evidence screened, assessed for eligibility, and included in the review, with reasons for exclusions at each stage, ideally using a flow diagram. | Page 10 (line 225-235) |
| Characteristics of sources of evidence | 15 | For each source of evidence, present characteristics for which data were charted and provide the citations. | Page 10 (line 237-252) |
| Critical appraisal within sources of evidence | 16 | If done, present data on critical appraisal of included sources of evidence (see item 12). | NA |
| Results of individual sources of evidence | 17 | For each included source of evidence, present the relevant data that were charted that relate to the review questions and objectives. | Page 11-12 (line 254-304) |
| Synthesis of results | 18 | Summarize and/or present the charting results as they relate to the review questions and objectives. | Page 10-12 (line 254-265; 277-281; 291-296) |
| **DISCUSSION** | | | |
| Summary of evidence | 19 | Summarize the main results (including an overview of concepts, themes, and types of evidence available), link to the review questions and objectives, and consider the relevance to key groups. | Page 12-17 (line 308-426) |
| Limitations | 20 | Discuss the limitations of the scoping review process. | Page 17 (line 428-440) |
| Conclusions | 21 | Provide a general interpretation of the results with respect to the review questions and objectives, as well as potential implications and/or next steps. | Page 18-19 (line 442-483) |
| **FUNDING** | | | |
| Funding | 22 | Describe sources of funding for the included sources of evidence, as well as sources of funding for the scoping review. Describe the role of the funders of the scoping review. | Page 19 (line 485-489) |

Legend: This table presents the PRISMA-ScR checklist items addressed in the scoping review, including the corresponding section, item number, item description, and the location in the manuscript (page and line numbers) where each item is reported. Items marked as "NA" indicate that they were not applicable to the review.

**Supplementary Table 2-** Search Strategy Used in Each Database for the Scoping Review

| **Database** | **Descriptors** | **Date (Day of April 2024)** | **Number of articles retrieved** |
| --- | --- | --- | --- |
| **Pubmed** | **“Sarcopenic obesity”** | **16** | **1360** |
| **Embase** | **“Sarcopenic obesity”** | **16** | **2472** |
| **LILACS** | **“Sarcopenic obesity” OR “Obesidade sarcopenica” OR “Obesidad sarcopénica”** | **13** | **29** |
| **Web of science** | **“Sarcopenic obesity”** | **13** | **2129** |
| **Scopus** | **“Sarcopenic obesity”** | **13** | **1851** |
| **MedRxiv** | **“Sarcopenic obesity”** | **13** | **33** |
| **Google scholar** | **“Sarcopenic obesity”** | **13** | **27500 (only considered the first 100 articles)** |
| **EBSCO** | **“Sarcopenic obesity”** | **13** | **2707 (1720 no duplicates)**  **MEDLINE Complete (1354)**  **Academic Search Premier (595)**  **CINAHL with Full Text (571)**  **SPORTDiscus with Full Text (72)**  **AgeLine (59)**  **Rehabilitation & Sports Medicine Source (38)**  **SocINDEX with Full Text (8)**  **Dentistry & Oral Sciences Source (5)**  **Business Source Complete (4)**  **Computers & Applied Sciences Complete (1)** |
| **BVS** | **“Sarcopenic obesity”** | **13** | **1637**  **MEDLINE (1522)**  **WPRIM (Pacífico Ocidental) (56)**  **LILACS (36)**  **IBECS (17)**  **CUMED (2)**  **Sec. Est. Saúde SP (2)**  **BBO - Odontologia (1)**  **BDENF - Enfermagem (1)**  **BINACIS (1)**  **LIPECS (1)**  **medRxiv (1)** |
| **Cochrane Central Register of Controlled Trial** | **“Sarcopenic obesity”** | **13** | **143** |
| **Physiotherapy Evidence Database** | **“Sarcopenic obesity”** | **13** | **31** |

**Legend:** Number of articles retrieved per database using the descriptor “Sarcopenic obesity” in April 2024. For each platform, the search date and total number of articles retrieved are presented. For databases integrated with multiple sources (e.g., EBSCO and BVS), results are shown both in total and by subdatabase. For Google Scholar, only the first 100 results were considered for screening due to volume.
